# Diffusion‐Driven Targeted Passivation of Selenium Vacancies via an I‐Doped CdS Buffer Layer for Efficient Sb_2_Se_3_ Solar Cells

**DOI:** 10.1002/advs.74890

**Published:** 2026-03-20

**Authors:** Luyan Shen, Deyang Qin, Er Nie, Jiahua Tao, Hidefumi Akiyama, Junhao Chu, Shaoqiang Chen

**Affiliations:** ^1^ Engineering Research Centre For Nanophotonics and Advanced Instrument School of Physics and Electronic Science East China Normal University Shanghai China; ^2^ State Key Laboratory of Precision Spectroscopy Department of Electronic Engineering East China Normal University Shanghai China; ^3^ Institute For Solid State Physics The University of Tokyo Kashiwa Chiba Japan

**Keywords:** defect passivation, iodine doped CdS, Ion diffusion, Sb_2_Se_3_ thin films, solar cells

## Abstract

Antimony selenide (Sb_2_Se_3_) is an emerging photovoltaic absorber with attractive optoelectronic properties, but its device performance is largely constrained by deep‐level defects, particularly selenium vacancies (V_Se_), which induce severe non‐radiative recombination. Herein, we report a targeted anion‐defect passivation strategy by incorporating iodine into the CdS buffer layer (CdS:I). Iodine doping not only promotes the preferential (100)‐oriented growth of CdS and passivates its intrinsic sulfur vacancies but also facilitates the spontaneous diffusion of iodine into the Sb_2_Se_3_ absorber during annealing. Density functional theory (DFT) and deep‐level transient spectroscopy (DLTS) analyses confirm that the diffused iodine atoms preferentially occupy V_Se_ sites, forming charge‐neutral I_Se_ defects with low formation energy. This results in a remarkable reduction in the defect capture cross‐section to 4.88 × 10^−22^ cm^2^. The optimized Sb_2_Se_3_ solar cell achieves a high open‐circuit voltage of 489.36 mV and a power conversion efficiency of 10.07%. This work provides an effective and generalizable defect‐passivation route for enhancing the performance of Sb_2_Se_3_ and related polycrystalline photovoltaic devices.

## Graphical abstract

Iodine incorporation into the CdS buffer layer induces spontaneous anion diffusion and selectively passivates selenium vacancies in Sb_2_Se_3_ absorbers. The formation of low‐energy, charge‐neutral I_Se_ defects effectively suppresses non‐radiative recombination, resulting in a substantially enhanced open‐circuit voltage and boosting the device efficiency to 10.07%.

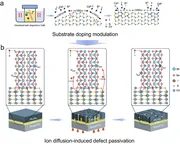

## 1 Introduction

Antimony selenide (Sb_2_Se_3_) is considered a highly promising light‐absorbing material for solar cells due to its suitable optical bandgap (1.17 eV), high absorption coefficient(≈10^5^ cm^1^), low toxicity, abundant elemental reserves, and good stability [1–4]. Sb_2_Se_3_ thin films are intrinsically p‐type due to acceptor defects such as antimony vacancies (V_Sb_) and antimony‐selenium antisites (Sb_Se_). Donor defects, including selenium vacancies (V_Se_) and selenium‐antimony antisites (Se_Sb_), act as deep‐level traps and can partially compensate the acceptor defects, modulated carrier concentration, and enhancing non‐radiative recombination. The material's quasi‐1D crystal structure and low lattice symmetry facilitate the facile formation of these defects in the bulk and at the heterojunction interface, highlighting the importance of defect balance for device performance [5, 6]. These defects have a large carrier capture cross section and will induce significant trap‐assisted Shockley‐Read‐Hall (SRH) non‐radiative recombination, resulting in severe open‐circuit voltage (V_OC_) loss, making the actual device efficiency far below the Shockley‐Queisser theoretical limit [7–9]. Therefore, suppressing donor state defects of Sb_2_Se_3_ thin films is crucial to improving device performance.

To suppress detrimental defects in Sb_2_Se_3_ thin films, researchers have explored strategies from multiple perspectives. Regarding preparation processes, optimizing the growth environment (e.g., by introducing additional selenium sources like selenourea), using auxiliary additives in chemical bath deposition, or adjusting the Sb/Se ratio in physical vapor deposition can moderately modulate thin‐film growth kinetics and inhibit defect formation [10–12]. Defect suppression by changing experimental conditions often lacks purpose in experimental design, and the result is mainly the passivation of defect energy levels and defect density, which actually corrects the growth mechanism of the film and improves the orderliness of the atomic chain arrangement. Such growth environmental adjustments lack precision in targeting specific deep‐level defects. In terms of interface engineering, promoting the diffusion of cations (e.g., Cd^2+^, Li^+^) at the heterojunction interface has been shown to improve vacancy defects at the interface through atomic radius selectivity, reduce carrier losses in the carrier separation stage, and thus improve the V_OC_ of the device [13–15]. However, due to charge mismatch, the introduced cations often generate new defect energy levels, which limit the accuracy and versatility of the passivation effect. Existing post‐deposition treatments (e.g., the use of reagents such as P_2_O_5_ and B_2_O_3_) have been used to suppress the escape of selenium and passivate selenium vacancies, which also belong to correcting atomic ratio offsets and reducing defect density [16, 17]. Therefore, developing a strategy that can selectively passivate deep energy level defects without introducing additional recombination centers remains a key challenge.

This study proposes an approach for the precise passivation of defects in the Sb_2_Se_3_ absorber layer by using I‐doped CdS buffer layer. It was found that I ions doping not only effectively induces preferential growth of CdS along the non‐polar (100) crystal plane but also suppresses its intrinsic sulfur vacancy defects. More importantly, during subsequent heat treatment, iodine ions spontaneously diffuse into the Sb_2_Se_3_ absorber layer. This thermally induced diffusion leads to the formation of I_Se_ neutral defects with very low formation energy, which effectively occupy selenium vacancy sites, achieving precise passivation and reducing the defect capture cross‐section significantly to 4.88 × 10^−22^ cm^2^. Benefiting from this defect passivation strategy, the Sb_2_Se_3_ solar cell achieved a high open‐circuit voltage of 489.36 mV and a final power conversion efficiency of 10.07%.

## 2 Results and Discussions

### 2.1 The Characterization of the I‐Doped CdS Buffer Layer

**FIGURE 1:**
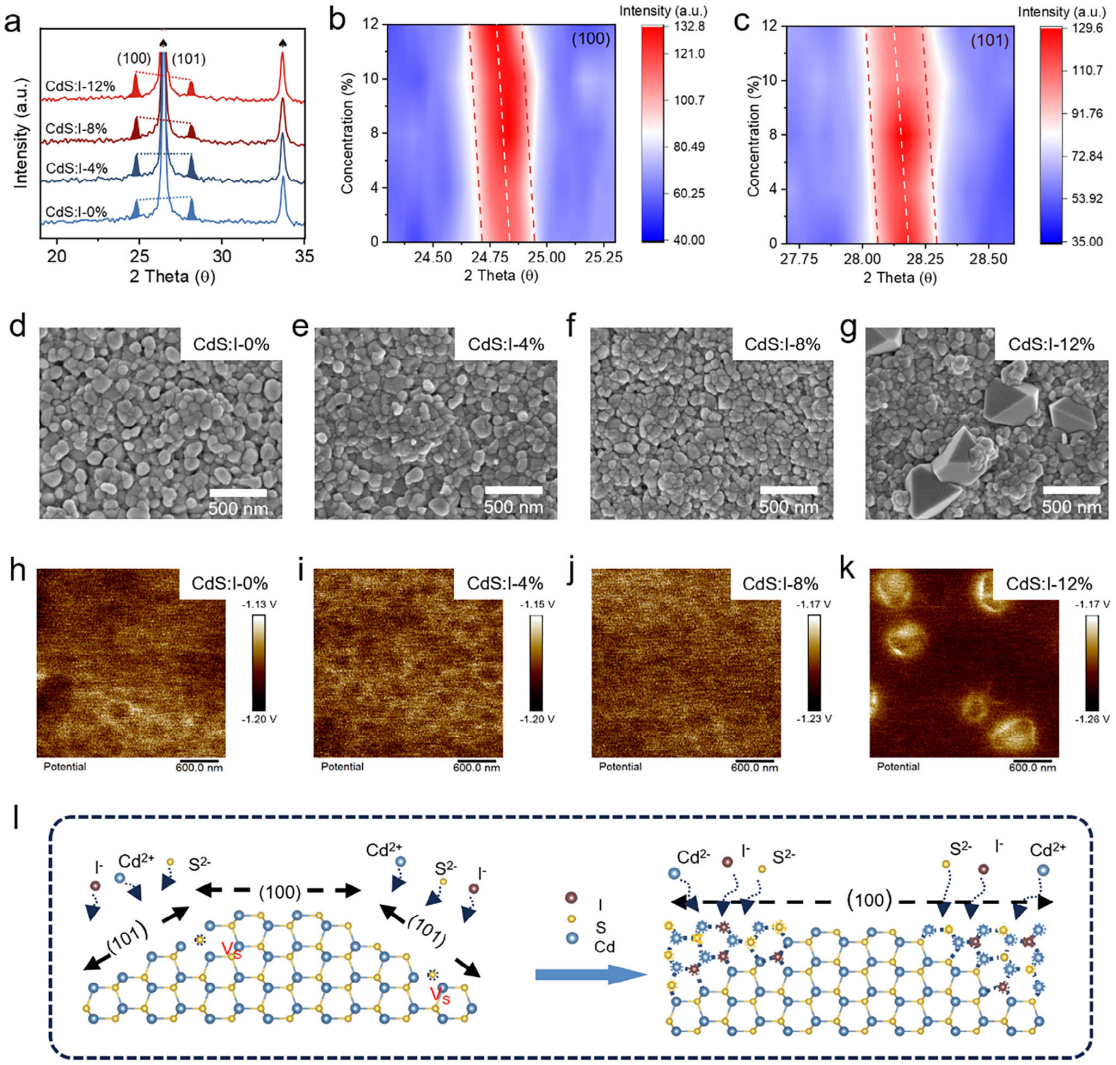
(a) XRD pattern of CdS films doped with different iodine contents (the peaks of FTO marked as ♠). (b,c) Contour map of the characteristic peak positions of CdS (100) and (101) crystal planes as a function of Iodine doping concentration. (d–g) SEM images of CdS:I‐0%, CdS:I‐4%, CdS:I‐8% and CdS:I‐12%. (h–k) The surface potential differences of CdS:I‐0%, CdS:I‐4%, CdS:I‐8% and CdS:I‐12%. (l) The crystal growth model of iodine‐doped CdS thin film. Iodine doping promotes preferential growth of the (100) crystal plane of CdS, while suppressing the growth of the (101) plane.

The crystal structure of CdS thin films was characterized by X‐ray diffraction (XRD). From the XRD patterns (Figure 1a), it can be observed that all the CdS films exhibit a hexagonal structure (JCPDS #41‐1049), with distinct (100) and (101) diffraction peaks (the peaks of FTO marked as, Figure S1) [18–20]. As the contents of I dopant concentration increases, the diffraction intensity of the (100) crystal plane is significantly enhanced, indicating that I doping effectively promotes the preferential growth of CdS along the [100] direction. Furthermore, the primary diffraction peaks of the (100) and (101) planes showed a consistent and systematic shift toward lower angles (Figure 1b,c). According to Bragg's law (nλ = 2d sinθ), this leftward shift in peak positions directly indicates an increase in the interplanar spacing (d). This strongly confirms that the larger iodide ions successfully substituted the smaller sulfide ions in the crystal lattice, resulting in anisotropic lattice expansion [21]. Scanning electron microscopy (SEM) further revealed that the film surface became progressively smoother with increasing iodine doping concentration; however, excessive iodine addition led to the formation of regular octahedral grains (Figure 1d–g). Further KPFM characterization was performed on I‐doped CdS surfaces with varying doping concentrations. The surface roughness values (Ra) for CdS:I‐0%, CdS:I‐4%, CdS:I‐8%, and CdS:I‐12% were measured as 22.8, 21.1, 18.8, and 35.7 nm, respectively, showing an initial decrease followed by an increase at higher doping levels (Figure S2). In contrast, the corresponding surface potentials remained largely unchanged, at −1.17, −1.18, −1.20, and −1.20 V, respectively (Figure 1h–k). These results clearly indicated that iodine doping primarily improved the film growth kinetics and mechanism, leading to optimized surface morphology, especially reduced roughness at moderate doping, without significantly altering the surface electrical potential landscape.

The structural and morphological evolution of CdS thin films reveals that I doping serves as an effective modulator of crystal growth kinetics (Figure 1l). High‐surface‐energy facets, such as the (101) plane, feature a higher density of undercoordinated atoms and active sites. Owing to their relatively large ionic radius and high electronegativity, I ions preferentially adsorb on these facets, thereby lowering the surface free energy of the CdS layer and potentially modulating the nucleation and growth of the Sb_2_Se_3_ thin films. This adsorption markedly lowers the surface energy of these planes and suppresses their growth rates, thereby promoting growth along the thermodynamically more stable (100) plane. Consequently, this orientation gradually becomes dominant [22–24]. Furthermore, iodine ions effectively passivate heterogeneous nucleation sites on the film surface, leading to a more uniform grain size distribution (Figure S3) [25]. This adsorption‐mediated competitive growth mechanism ultimately leads to the enhancement of the (100) orientation in the CdS thin films. This oriented growth not only refines the film microstructure but is also expected to influence charge transport and interfacial properties, which are critical for optoelectronic applications.

**FIGURE 2:**
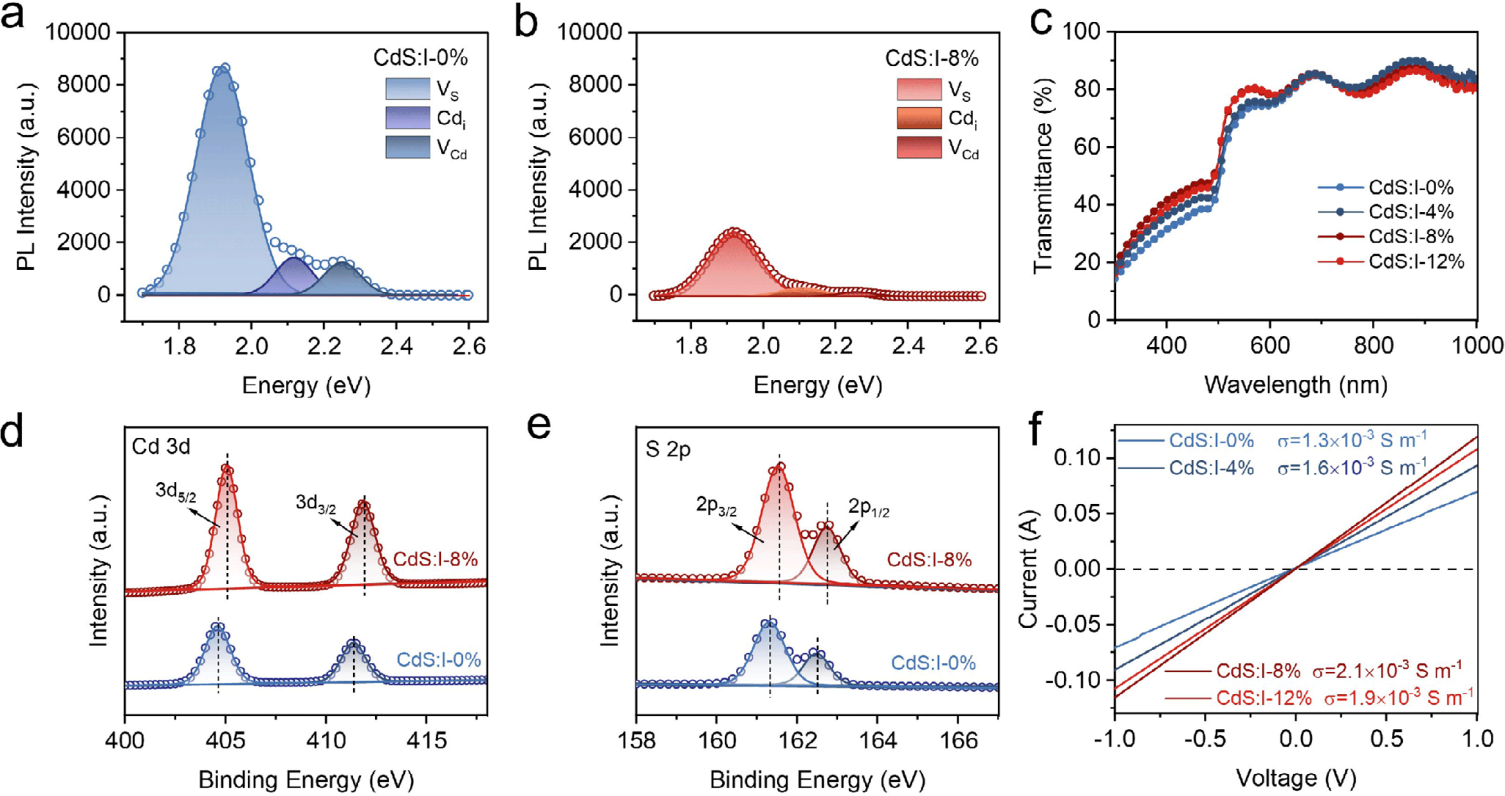
PL spectra of (a) CdS:I‐0% and (b) CdS:I‐8% films. (c) The optical transmittance spectra of CdS with different concentration I‐doping. X‐ray photoelectron spectroscopy (XPS) of (d) Cd 3d and (e) S 2p for the CdS:I‐0% and CdS:I‐8% films, respectively. (f) *I‐V* characteristics of the FTO/ CdS:I‐x%/Au devices.

Photoluminescence (PL) spectroscopy confirms a significant suppression of defect states (Figure 2a,b; Figure S4). All CdS samples exhibit characteristic emission peaks at 1.91, 2.14, and 2.25 eV, which correspond to sulfur vacancies (V_S_), cadmium interstitials (Cd_i_), and cadmium vacancies (V_Cd_), respectively [26, 27]. Such defects are difficult to avoid completely during the CBD growth of CdS. The undoped sample shows a broad defect‐related emission band around 1.91 eV, originating mainly from non‐radiative recombination. After iodine doping, the intensity of this emission band decreases markedly by about 73%, indicating that iodine effectively passivates non‐radiative recombination centers such as sulfur vacancies, thereby substantially reducing defect‐induced carrier recombination losses. The distinct I 3d peak visible in the X‐ray photoelectron spectroscopy (XPS) spectrum (Figure S5a) at a binding energy of 618 eV confirms the successful incorporation of iodine into the CdS lattice [28]. Furthermore, no sodium ions were detected on the sample surface, confirming that sodium ions were effectively removed during the cleaning process (Figure S5b). More importantly, compared to the undoped sample, both the Cd 3d and S 2p peaks in the I‐doped sample exhibit a systematic positive shift in binding energy (Figure 2d,e), indicating that the introduction of I modifies the electronic environment of the Cd─S bonds, effectively passivating V_S_ defects and mitigating the charge deficiency at sulfur sites [29, 30]. The passivation of V_S_ defects by I significantly improves the electrical properties of the CdS thin films, which is expected to enhance electron extraction efficiency and overall device performance. As shown in the optical transmission spectrum (Figure 2c), the CdS:I film exhibits higher transmittance across the wavelength range of 300–550 nm compared to the undoped CdS. This reduces parasitic absorption in the buffer layer, allowing more incident light to reach the Sb_2_Se_3_ absorber. Consequently, the light harvesting in Sb_2_Se_3_ is improved, which contributes directly to the increased short‐circuit current density (J_SC_) of the device. Furthermore, the electrical conductivity of the film increased from 1.3 × 10^−3^ S m^−1^ for the undoped sample to 2.1 × 10^−3^ S m^−1^ after I‐8% doping (Figure 2f; Figure S6).

### 2.2 I‐Ion Diffusion Mechanism in Sb_2_Se_3_ Thin Films

**FIGURE 3:**
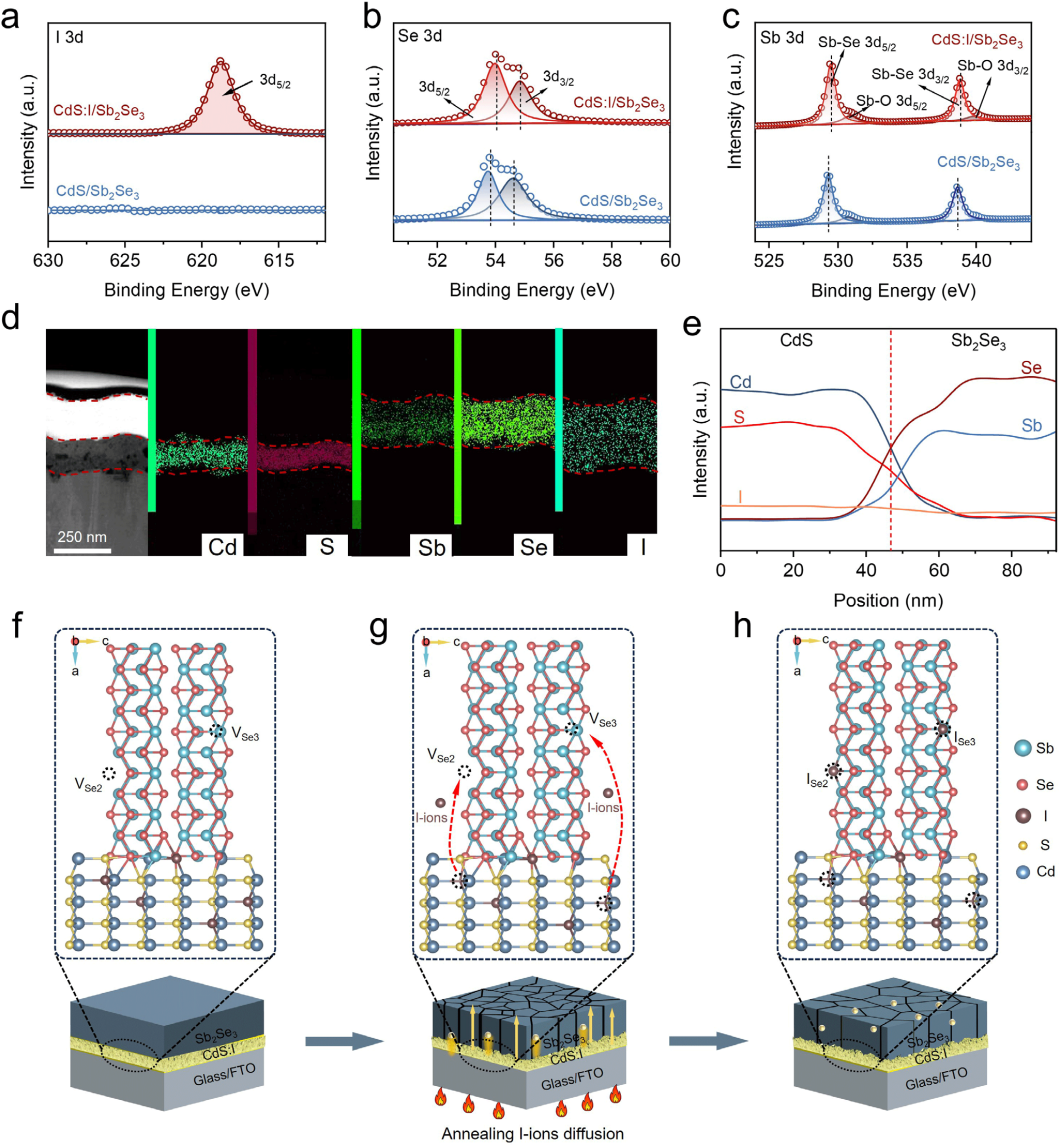
The XPS of (a) I 3d, (b) Se 3d, and (c) Sd 3d for the CdS/Sb_2_Se_3_ and CdS:I/Sb_2_Se_3_ films, respectively. (d) The cross‐sectional EDS elemental mapping of CdS:I/Sb_2_Se_3_ device. (e) EDS elemental line scan profiles across the CdS:I/Sb_2_Se_3_ section. (f–h) Schematic diagram of the diffusion of I‐ions from the CdS film to the Sb_2_Se_3_.

The Sb_2_Se_3_ thin films were prepared by CBD and subsequently annealed at 375°C for 5 min under N_2_ atmosphere. To probe the chemical bond change upon the I‐doping, we conducted XPS characterizations. In the XPS spectrum of CdS:I/Sb_2_Se_3_ thin films, a prominent characteristic peak can be observed at 618.77 eV, which corresponds to the I 3d_5/2_ energy level (Figure 3a) [31]. In the Se 3d spectrum of the CdS/Sb_2_Se_3_ sample, peaks at binding energies of 53.7 and 54.6 eV are assigned to Se 3d_5/2_ and Se 3d_3/2_, respectively (Figure 3b). Notably, in the CdS:I/Sb_2_Se_3_ sample, these Se characteristic peaks shift slightly to higher binding energies, moving to 54.0 and 54.8 eV. Similarly, in the Sb 3d spectrum, the CdS/Sb_2_Se_3_ sample shows peaks at 529.28 and 538.64 eV, which can be assigned to Sb 3d_5/2_ and Sb 3d_3/2_, respectively, in CdS:I/Sb_2_Se_3_, these peaks also shift to 529.47 and 538.84 eV (Figure 3c) [32]. These systematic shifts in binding energy are primarily attributed to the changes in the local chemical environment caused by iodine doping. Due to its higher electronegativity, iodine incorporation induces a redistribution of valence electrons toward the iodine sites, which reduces the effective electronic screening around the Sb and Se atomic nuclei. The weakened screening enhances the Coulomb interaction between the electrons and the nuclei, resulting in an overall shift of the Sb 3d and Se 3d binding energies toward higher values. Furthermore, the longitudinal distribution of elements in the thin film was analyzed. Figure 3d shows the TEM cross‐section of the CdS:I/Sb_2_Se_3_ interface region and the corresponding EDS elemental mapping. The results indicate distinct boundaries for elements such as Cd, S, Se, and Sb at the interface, while I was uniformly distributed throughout both the CdS:I and Sb_2_Se_3_ layers. Local EDS line scanning analysis provided a more precise characterization of the elemental distribution near the heterojunction interface (Figure 3e; Figure S7). The interface between the electron transport layer (ETL) and the absorption layer is clearly distinguishable, and iodine is uniformly distributed in both layers, suggesting its diffusion and distribution throughout the structure during the annealing process.

Based on the aforementioned results, it can be inferred that during the annealing process, iodide ions diffuse from the CdS:I layer into the Sb_2_Se_3_ layer, a process primarily driven by the chemical potential gradient and facilitated by the low ionic migration energy barrier (Figure 3f,g). Due to their high electronegativity, once incorporated into the Sb_2_Se_3_ lattice, the iodide ions preferentially occupy selenium vacancies (V_Se_) while simultaneously attracting electron density from neighboring Se and Sb atoms (Figure 3h). This electron transfer increases the effective nuclear charge experienced by the outer electrons of Se and Sb, thereby enhancing their binding energies, a trend that aligns consistently with the XPS analysis. These results indicate that iodine not only diffuses into the Sb_2_Se_3_ film but also establishes weak chemical interactions with Se and Sb atoms.

**FIGURE 4:**
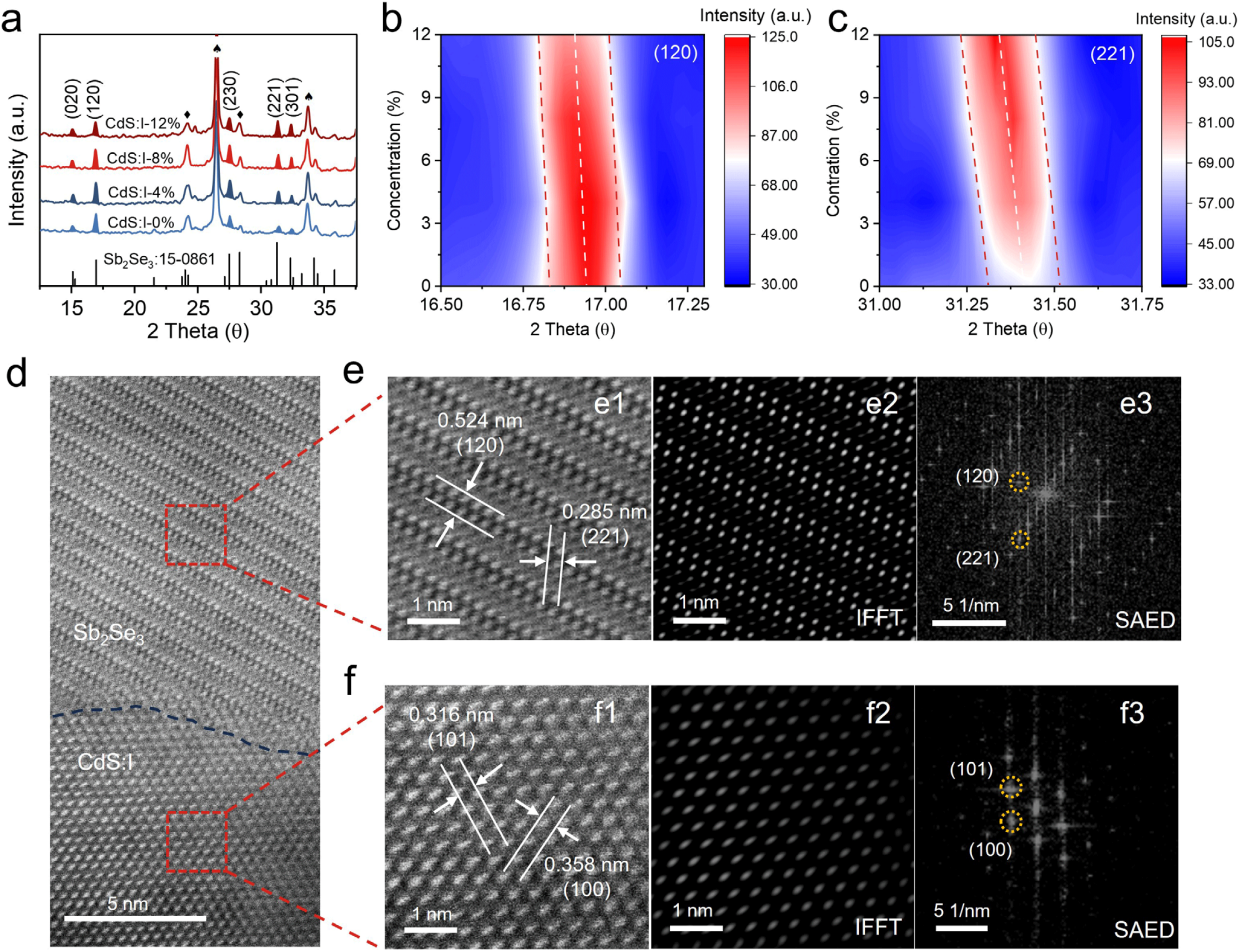
(a) XRD diffraction patterns of Sb_2_Se_3_ based on CdS:I‐x% (x from 0% to 12%). (b,c) Contour map of the characteristic peak positions of Sb_2_Se_3_ (120) and (221) crystal plane as a function of CdS:I‐x% (x from 0% to 12%). (d) HAADF‐STEM image of the CdS:I/Sb_2_Se_3_ heterojunction interface. (e1,f1) Corresponding HAADF‐STEM images acquired from the Sb_2_Se_3_ side and the CdS side, respectively. (e2,f2) The Inverse Fast Fourier transform (IFFT) images of the areas in (e1,f1). (e3,f3) Corresponding selected‐area electron diffraction (SAED) patterns of the areas in (e1,f1).

XRD characterization was conducted to investigate the crystal structure of the films. Figure 4a shows the XRD patterns of Sb_2_Se_3_ films deposited on CdS:I substrates with varying doping concentrations (x%), where the diffraction peaks corresponding to FTO and CdS are marked with ♠ and ♦, respectively. Several major characteristic peaks of the thin films are located at 15.05°, 16.9°, 27.50°, 31.34°, and 32.42°, which correspond to the (020), (120), (230), (221), and (301) crystal planes of Sb_2_Se_3_ (JCPDF #15‐0861), respectively. Furthermore, the texture coefficient (TC) of these films was calculated. The preferred growth of Sb_2_Se_3_ films along the substrate‐normal (ℎ𝑘1) planes is enhanced with higher I dopant concentrations in CdS (Figure S8). This enhanced [ℎ𝑘1]‐oriented growth is mainly attributed to the template effect of non‐polar crystal planes in the CdS substrate [19, 20]. During this process, the [ℎ𝑘1] orientation is strengthened, while the [ℎ𝑘0] orientation is suppressed. Such an evolution in microstructure is expected to improve charge transport efficiency when the films are applied in solar cell devices. It is noteworthy that as the I dopant concentration in CdS increases, the diffraction peak corresponding to the (120) and (221) plane in the Sb_2_Se_3_ thin films gradually shifts toward lower angles, indicating that I ions may have diffused into the Sb_2_Se_3_ layer during annealing and promoted the formation of a novel I‐doped compound. (Figure 4b,c). The crystal structure and heterojunction interface properties between Sb_2_Se_3_ and CdS:I were further investigated using high‐angle annular dark‐field scanning transmission electron microscopy (HAADF‐STEM) (Figure 4d). The observations revealed a clear and high‐quality heterojunction interface with no significant defects such as lattice distortions, dislocations, or amorphous layers. Figure 4e presents a magnified local image of the Sb_2_Se_3_ region (Figure 4e1), the corresponding inverse fast Fourier transform (IFFT) pattern (Figure 4e2), and the selected area electron diffraction (SAED) pattern (Figure 4e3). Lattice fringes with spacings of 0.524 and 0.287 nm were clearly identified, corresponding to the (120) and (221) crystal planes of orthorhombic Sb_2_Se_3_, respectively. Diffraction spots consistent with the (120) and (221) planes were also observed in the SAED pattern, which aligns with the XRD analysis. Similarly, corresponding analysis was conducted on the CdS:I region, with Figure 4f1–f3 displaying the magnified local image, IFFT pattern, and SAED pattern of this region, respectively. In Figure 4f1, lattice fringes with spacings of 0.358 and 0.316 nm were observed, corresponding to the (100) and (101) crystal planes of CdS, indicating a polycrystalline structure. Diffraction spots of the (100) and (101) planes were also clearly visible in the SAED pattern. All these crystalline structural features are consistent with the XRD results.

### 2.3 Defect Analysis

**FIGURE 5:**
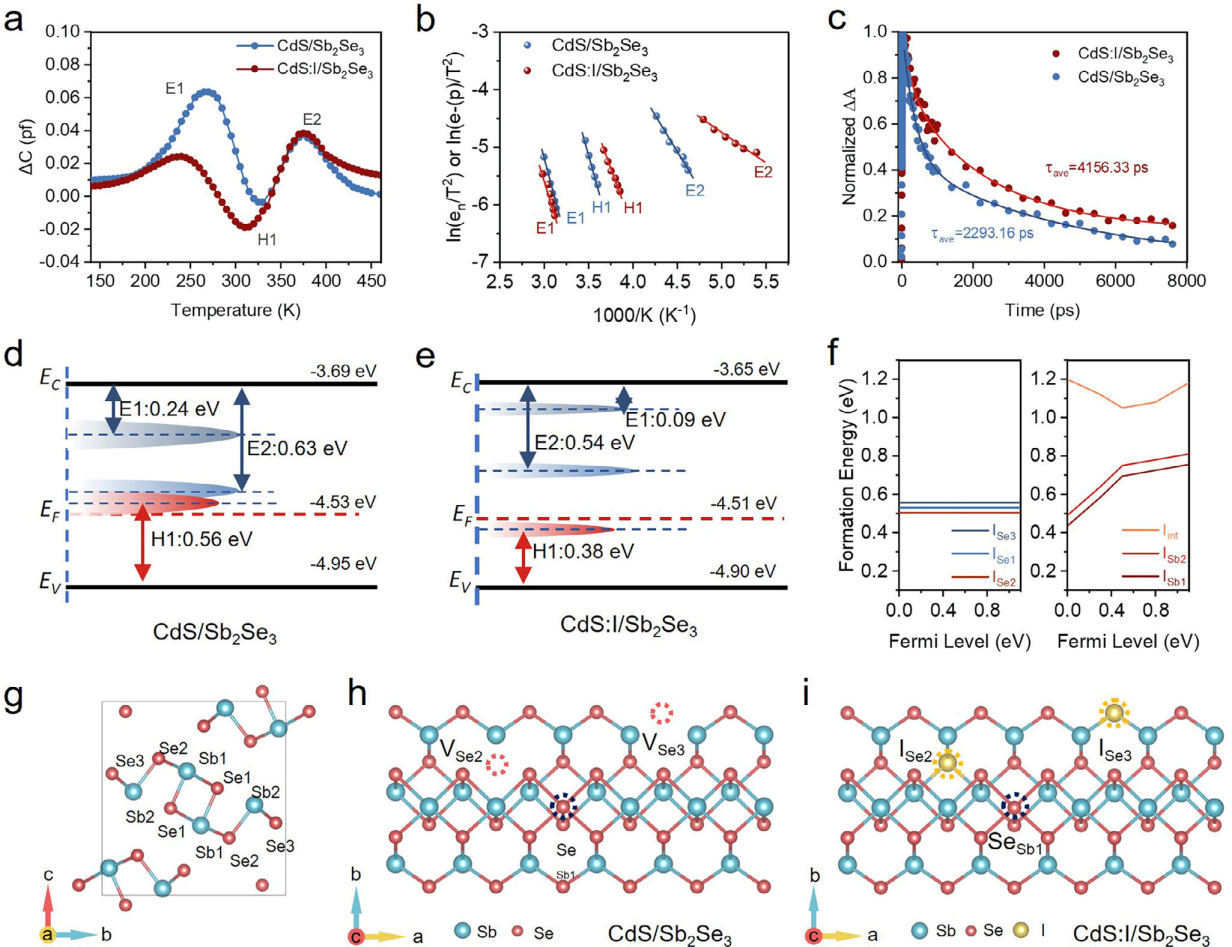
(a) The DLTS single pattern of CdS/Sb_2_Se_3_ and CdS:I/Sb_2_Se_3_ films devices. (b) Arrhenius plots for the CdS/Sb_2_Se_3_ and CdS:I/Sb_2_Se_3_ films devices. (c) Transient decay kinetics and fitting curves of the CdS/Sb_2_Se_3_ and CdS:I/Sb_2_Se_3_ films, respectively. (d,e) The energy levels and defect levels of the CdS/Sb_2_Se_3_ and CdS:I/Sb_2_Se_3_ film device. (f) The formation energy vs. Fermi level plots of CdS:I/Sb_2_Se_3_ calculated by density functional theory (DFT). (g) Sb_2_Se_3_ crystal structure viewed from the a‐direction. (h) Defects positions of V_Se2_, V_Se3_, and Se_Sb1_ in the CdS:I/Sb_2_Se_3_ structure. (i) Defects positions of I_Se2_ and I_Se3_ and Se_Sb1_ in the CdS:I/Sb_2_Se_3_ structure.

The defect characteristics of CdS/Sb_2_Se_3_ and CdS:I/Sb_2_Se_3_ films were investigated by using the deep‐level transient spectroscopy (DLTS). The DLTS signal spectra and Arrhenius plots for CdS/Sb_2_Se_3_ and CdS:I/Sb_2_Se_3_ solar cell are shown in Figure 5a,b, respectively. Distinct positive and negative peaks in the spectra correspond to electron traps and hole traps [33]. One hole trap (H1) and two electron traps (E1 and E2) were identified in both samples. Detailed defect parameters, including energy levels (E_T_), capture cross‐sections (σ), and defect densities (N_T_), are summarized in Table 1. To better evaluate the correlation between defects and device performance, the bandgap of the Sb_2_Se_3_ film was determined by UV–vis spectroscopy, and the conduction band (E_C_), valence band (E_V_), and Fermi level (E_f_) positions were obtained from ultraviolet photoelectron spectroscopy (UPS) (Figure S9).

**TABLE 1:** The details defect information obtained from DLTS characterizations of CdS/Sb_2_Se_3_ and CdS:I/Sb_2_Se_3_ devices.

Devices	Defect	E_T_ (eV)	σ (cm^2^)	N_T_ (cm^−3^)
CdS/Sb_2_Se_3_	E1 (V_Se2_)	E_C_‐0.24	3.43 × 10^−19^	7.96 × 10^15^
	H1 (Se_Sb1_)	E_v_+0.56	1.52 × 10^−15^	2.75 × 10^14^
	E2 (V_Se3_)	E_C_‐0.63	1.23 × 10^−15^	4.63 × 10^15^
CdS:I/Sb_2_Se_3_	E1 (V_Se2_)	E_C_‐0.097	4.88 × 10^−22^	1.18 ×10^15^
	H1 (Se_Sb1_)	E_V_+0.38	3.20 × 10^−18^	5.8 × 10^14^
	E2 (V_Se3_)	E_C_‐0.54	2.09 × 10^−17^	2.85 × 10^15^

In the CdS/Sb_2_Se_3_ sample, the defect levels of E1, H1, and E2 were measured at 0.24, 0.56, and 0.63 eV, respectively (Figure 5d). In contrast, these levels shifted significantly to 0.097, 0.38, and 0.54 eV in the CdS:I/Sb_2_Se_3_ sample (Figure 5e). Based on previous DFT calculations, E1, H1, and E2 are attributed to Se2 vacancy (V_Se2_), Se_Sb_ antisite (Se_Sb1_), and Se3 vacancy (V_Se3_) defect, respectively [34, 35, 6, 8]. V_Se3_ are donor‐type defects that introduce electrons and neutralize the p‐type doping of the film itself, reducing the carrier transport efficiency. Moreover, the deep‐level defects V_Se3_ and Se_Sb1_ strongly enhance Shockley‐Read‐Hall (SRH) recombination and cause Fermi‐level pinning, thereby degrading carrier transport and photovoltaic performance. In addition, the capture cross‐sections of the E1, H1, and E2 defects were reduced by two to three orders of magnitude, declining from 3.43 × 10^−19^, 1.52 × 10^−15^, and 1.23 × 10^−15^ cm^2^ in the CdS/Sb_2_Se_3_ film to 4.88 × 10^−22^, 3.20 × 10^−18^, and 2.09×10^−17^ cm^2^ in the CdS:I/Sb_2_Se_3_ film. The simultaneous reduction in the E_T_ and σ of the E1, H1, and E2 defects indicates a significantly weakened ability of these defects to capture charge carriers in CdS:I/Sb_2_Se_3_, thereby reducing the probability of charge recombination. This provides clear evidence that the diffusion of I into CdS:I/Sb_2_Se_3_ effectively passivates the deep‐level defects within the Sb_2_Se_3_ layer, contributing to the suppression of non‐radiative recombination processes.

Further studies on the carrier transport dynamics of CdS/Sb_2_Se_3_ and CdS:I/Sb_2_Se_3_ using transient absorption spectroscopy (TAS) were conducted. As shown in Figure S10, a distinct photo‐induced absorption (PIA) peak at ∼705 nm appears in both samples, attributed to the trapping of photogenerated minority carriers. The kinetic decay curves were fitted with a biexponential model (Figure 5c), with detailed parameters listed in Table S1. Specifically, the short lifetime component (τ_1_) and the long one (τ_2_) correspond to interface/surface recombination and bulk defect recombination, respectively. Compared to the control CdS/Sb_2_Se_3_, the CdS:I/Sb_2_Se_3_ film exhibits a significant increase in both τ_1_ and τ_2_, indicating suppressed recombination at both the interface and in the bulk, with the latter being the dominant mechanism. This conclusion aligns with the DLTS results. The calculated average decay lifetimes are 2293.16 ps for CdS/Sb_2_Se_3_ and 4156.33 ps for CdS:I/Sb_2_Se_3_. Overall, the suppression of deep‐level defects in the Sb_2_Se_3_ absorber, the improved junction quality, and the extended carrier lifetime collectively contribute to enhancing the V_OC_ and PCE of the corresponding thin‐film solar cells.

To further clarify the passivation mechanism, we performed density functional theory (DFT) calculations on I‐doped Sb_2_Se_3_ (Figure 5f). It is noteworthy that the I_Se_ defect has low formation energy, and it remains nearly constant across the entire Fermi level range (from VBM to CBM), with a slope close to zero. This theoretically indicates that the I_Se_ defect maintains a fixed charge state under the considered thermodynamic conditions. This behavior can be attributed to the near‐isovalent substitution characteristic of iodine for selenium, along with the resulting defect energy levels being located near the band edges or within the bands, which prevents any charge‐state transition within the bandgap. This stands in sharp contrast to the behavior of the intrinsic selenium vacancy (V_Se_), whose formation energy exhibits significant Fermi‐level dependence, corresponding to its multiple charge states. Therefore, the I_Se_ defect is not only thermodynamically favorable but also electronically stable, and its passivation effect is not easily influenced by fluctuations in the Fermi level. This provides microscopic mechanistic support for the stable enhancement of device performance achieved through iodine doping. This explains the absence of new defect levels in the DLTS spectra and further confirms that iodine diffusion effectively passivates deep‐level defects in Sb_2_Se_3_ films (Figure 5g–i).

To evaluate the reduction in trap state density, we employed the space charge limited current (SCLC) model to test both the CdS/Sb_2_Se_3_ and CdS:I/Sb_2_Se_3_ samples [36, 37]. The trap‐filling limit voltage (V_TFL_) for CdS:I/Sb_2_Se_3_ is 0.15 V, compared to 0.29 V for CdS/Sb_2_Se_3_ (Figure S11). The lower V_TFL_ value indicates a significant reduction in trap state density, consistent with theoretical expectations. The calculated electron trap density in CdS:I/Sb_2_Se_3_ is 3.45 × 10^15^ cm^−3^, which is lower than that of CdS/Sb_2_Se_3_ (6.67 × 10^15^ cm^−3^). The reduction in total trap density measured by SCLC is consistent with the specific defect passivation observed via DLTS. While DLTS identifies and quantifies discrete deep levels, SCLC provides an integrated measure of all electron‐accessible traps. The combined density of the primary electron traps identified by DLTS (E1/V_Se2_ and E2/V_Se3_ in CdS:I/Sb_2_Se_3_, totaling ∼4.0 × 10^15^ cm^−3^) aligns well in order of magnitude with the total trap density from SCLC (3.45 × 10^15^ cm^−3^). This agreement confirms that the passivation of the specific deep‐level defects V_Se2_ and V_Se3_ is the primary driver for the overall reduction in charge‐trapping states, leading to suppressed non‐radiative recombination.

### 2.4 Device Performance and Characterization

**FIGURE 6:**
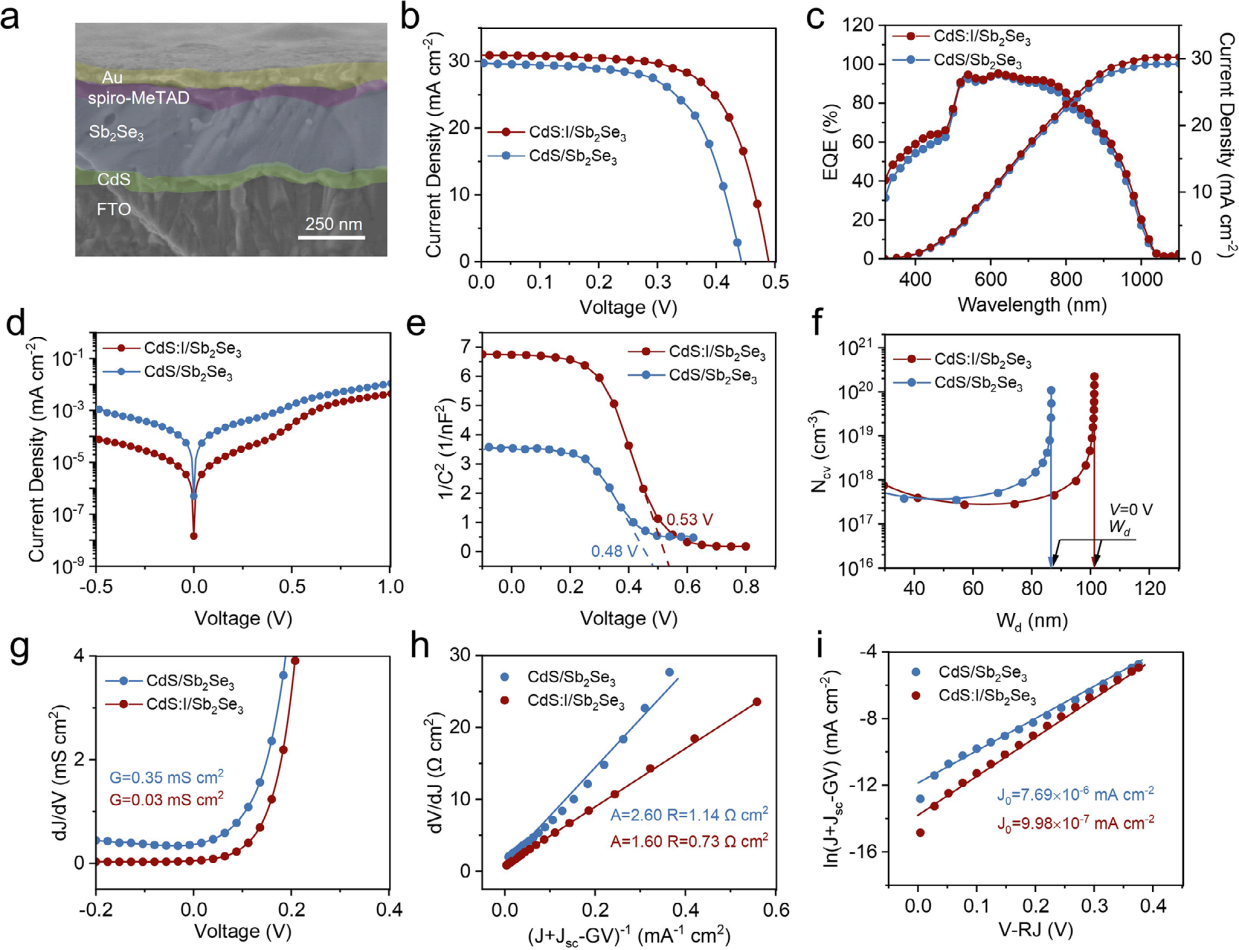
(a) The cross‐sectional SEM image of a typical Sb_2_Se_3_ planar heterojunction solar cell. The (b) *J‐V* curves and (c) EQE spectra for CdS/Sb_2_Se_3_ solar cell and CdS:I/Sb_2_Se_3_ solar cell. (d) Semi‐logarithmic dark J‐V plots, (e) Mott‐Schottky plots (1/C^2^‐V), (f) N_C‐V_ curves of CdS/Sb_2_Se_3_ solar cell and CdS:I/Sb_2_Se_3_ solar cell. (g) dJ/dV vs. Voltage, (h) dV/dJ vs. (J+J_SC_‐GV), and (i) ln(J+J_SC_‐GV)^−1^ vs. V‐RJ plots for CdS/Sb_2_Se_3_​ solar cells and CdS:I/Sb_2_Se_3_​ solar cells.

The device architecture was FTO/CdS:I‐x%/Sb_2_Se_3_/spiro‐OMeTAD/Au, with layer thicknesses of 69, 279, 82, and 90 nm from the CdS layer to the Au electrode, respectively (Figure 6a). To investigate the impact of I dopant concentration on device performance, a series of Sb_2_Se_3_ solar cells employing CdS:I buffer layers with different doping levels (x%) were fabricated (Figure S12). Device efficiency exhibits a non‐monotonic dependence on iodine doping concentration, peaking before a sharp decline as doping reaches 12%, marked by significant drops in both J_SC_ and FF. This degradation is directly attributed to the deterioration of the CdS:I buffer layer under excessive doping. Specifically, AFM and SEM morphology (Figure 1d–g; Figure S2) and the re‐emergence of defect‐related PL emission (Figure 2a,b; Figure S4) confirm that over doping introduces film inhomogeneity and new defect states. The resulting rough and defective buffer layer degrades the CdS/Sb_2_Se_3_ heterojunction quality, increasing interface recombination and series resistance. As a result, charge collection is impaired, leading to the concurrent reduction in J_SC_ and FF. A comparison of the current density‐voltage (*J‐V*) characteristics between the best‐performing CdS:I/Sb_2_Se_3_ device and the CdS/Sb_2_Se_3_ device is provided in Figure 6b. The CdS/Sb_2_Se_3_ device exhibited a V_OC_ of 443.33 mV, J_SC_ of 29.71 mA cm^−2^, FF of 62.52%, and PCE of 8.27%. In contrast, the CdS:I/Sb_2_Se_3_ device shows comprehensive improvement in all key photovoltaic parameters, with a V_OC_ of 489.36 mV, J_SC_ of 30.89 mA cm^−2^, FF of 66.66%, and resulting PCE of 10.07%, as summarized in Table 2, achieving a 21.7% performance improvement. In addition, the unencapsulated device retained 93.5% of its initial efficiency after 300 h of storage in a desiccator at ambient temperature and 20%±5% relative humidity, confirming its excellent stability (Figure S13). The J_SC_ values of all devices are confirmed by external quantum efficiency (EQE) measurements (Figure 6c). The response of the CdS:I/Sb_2_Se_3_ device in the spectral range of 300–550 nm is significantly enhanced due to the optimization of both the CdS buffer layer. The EQE‐integrated photocurrent densities are 29.23 and 30.25 mA cm^−2^ for the CdS/Sb_2_Se_3_ and CdS:I/Sb_2_Se_3_, respectively, in agreement with the J_SC_ values obtained from the *J‐V* curves.

**TABLE 2:** The photovoltaic parameters of CdS:I‐x%/Sb_2_Se_3_ solar cells.

Devices	V_OC_ (mV)	J_SC_ (mA cm^−2^)	FF (%)	PCE (%)
CdS:I‐0%/Sb_2_Se_3_	443.33	29.71	62.85	8.27
CdS:I‐4%/Sb_2_Se_3_	470.01	29.88	64.44	9.05
CdS:I‐8%/Sb_2_Se_3_	489.36	30.89	66.66	10.07
CdS:I‐12%/Sb_2_Se_3_	473.84	29.93	63.88	9.06

Notably, the significant increase in V_OC_ from 443.33 to 489.36 mV is identified as the primary factor contributing to the enhanced device efficiency. The increase in V_OC_ is attributed to the suppression of non‐radiative recombination within the device, particularly Shockley‐Read‐Hall (SRH) recombination associated with deep‐level defects [38–40]. This is further supported by the significantly reduced reverse saturation current (*J*_0_) observed in the dark *J‐V* measurements of the CdS:I devices, indicating a reduction in non‐radiative recombination pathways (Figure 6d) [41]. To elucidate the origin of the enhanced V_OC_, we further investigated the electrical properties of the devices. Mott‐Schottky (M‐S) analysis revealed a substantially increased built‐in potential (V_bi_) in the CdS‐device, which strengthens the driving force for charge separation and collection, a key factor contributing to higher V_OC_. Additionally, C‐V depth profiling (Figure 6e,f) shows an order‐of‐magnitude enhancement in the apparent carrier concentration (N_A_) of the Sb_2_Se_3_ absorber when using a CdS:I buffer layer (1.41 × 10^20^ cm^3^) vs. a CdS buffer (2.57 × 10^19^ cm^3^) [42]. We attribute this increase in N_A_ to the possible diffusion of iodine atoms into the Sb_2_Se_3_ lattice, where they effectively passivate deep‐level defects and reduce the self‐compensation effect in the antimony selenide, thereby enhancing the ionization efficiency of acceptors.

Electrochemical impedance spectroscopy (EIS) was employed to investigate the charge carrier recombination and transport properties. Nyquist plots of the two devices are shown in Figure S14, with the equivalent circuit model provided in the inset. The fitting results reveal that the CdS:I/Sb_2_Se_3_ device exhibits a larger recombination resistance (R_rec_ = 1258.6 Ω) and a smaller charge transfer resistance (R_s_ = 17.26 Ω), compared to the CdS/Sb_2_Se_3_ device with corresponding values of 991.4 and 41.53 Ω, respectively. This confirms that the use of CdS:I effectively suppresses interfacial recombination and thus enhances charge carrier transport efficiency. The enhancement of the built‐in electric field and the increase in carrier concentration synergistically drive the formation of a more robust p‐n junction and effectively suppress interfacial recombination, ultimately leading to a significant improvement in the V_OC_.

To further investigate the electrical characteristics underlying the enhanced device performance, we analyzed the dark current‐voltage characteristics of the fabricated solar cells (Figure 6g–i). Key performance parameters, including parallel conductance (G), reverse saturation current (*J*_0_), diode ideality factor (A), and series resistance (*R_S_*), were extracted using the following equation [43, 44]:

(1) $$J=J_{0}\;\exp\left[\frac{q}{Ak_{B}T}\left(V-JR_{s}\right)\right]+GV-J_{L}$$

The CdS:I/Sb_2_Se_3_ device exhibited a G of 0.03 mS cm^−2^, a *R_S_* of 0.73 Ω cm^2^, A of 1.60, and a *J*_0_ of 9.98 × 10^−7^ mA cm^−2^. All these parameters are superior to those of the control CdS/Sb_2_Se_3_ device, which showed a G of 0.35 mS cm^−2^, Rs of 1.14 Ω cm^2^, A of 2.60, and a *J*_0_ of 7.69 × 10^−6^ mA cm^−2^. The improved electrical properties of the CdS:I/Sb_2_Se_3_ device clearly correlate with its higher overall performance.

In summary, the enhancement in device performance originates from the synergistic improvement of both the CdS buffer layer and the Sb_2_Se_3_ absorber, mediated by iodine. While the fundamental optoelectronic properties of CdS, such as its optical bandgap and Fermi level position, remain largely unchanged (Figure 1h–k; Figure S15 and Table S2), its electrical quality is markedly improved. I dopant significantly increases the conductivity of CdS and effectively passivates its V_S_ defects (Figure 2a,b,f; Figure S4). This creates a more robust and electronically benign interface. Concurrently, iodine incorporation in Sb_2_Se_3_ suppresses deep‐level bulk defects, as confirmed by DLTS and TAS. The confluence of a high‐quality buffer and a high‐quality absorber minimizes recombination at the heterojunction interface and within the bulk, leading to more efficient charge collection. This comprehensive passivation strategy, targeting both interfacial and bulk defects, is the cornerstone of the observed improvements in V_OC_, J_SC_, FF, and PCE.

## 3 Conclusion

In summary, we have successfully developed an effective anion‐defect passivation strategy for Sb_2_Se_3_ solar cells by utilizing an I‐doped CdS buffer layer. The incorporation of iodine into CdS was found to induce favorable (100)‐oriented growth, improve film compactness, and passivate sulfur vacancies within the buffer layer itself. Crucially, post‐deposition annealing facilitated the diffusion of iodine into the Sb_2_Se_3_ absorber. Combined DLTS analysis and DFT calculations confirmed that iodine atoms preferentially occupy selenium vacancy sites (V_Se2_ and V_Se3_), forming benign I_Se_ defects that effectively neutralize these deep‐level recombination centers without introducing new trap states. This targeted passivation led to a drastic reduction in defect capture cross‐section and density, thereby significantly suppressing non‐radiative SRH recombination. As a result, the devices exhibited a substantial improvement in V_OC_ and overall efficiency, reaching a champion PCE of 10.07%. This work highlights the great potential of anion‐diffusion‐based defect engineering for overcoming the performance limitations of Sb_2_Se_3_ and other similar polycrystalline solar cells.

## 4 Experimental Section

### 4.1 Preparation of Electron Transport Layers (ETL)

First, FTO‐coated glass (AGC 2.2, Advanced Election Technology Co., Ltd.) was ultrasonically cleaned sequentially with deionized water, detergent, acetone, isopropanol, and ethanol for 20 min, followed by UV‐ozone treatment for 15 min for surface activation. CdS thin films were then deposited via chemical bath deposition (CBD): Under constant stirring at 68.5°C, a reaction mixture was prepared by combining 10 mL of 3 mol L^−^^1^ CdSO_4_ solution, 10 mL of 1.5 mol L^−^^1^ thiourea solution, 26 mL of 28% ammonia, and 154 mL of ultrapure water (total volume is 200 mL). Pre‐cleaned FTO substrates were immersed in this mixture. When pale yellow appeared after exactly 3 min of reaction, specified volumes of 0.03 mol L^−^^1^ NaI solution were added according to target n_I_/n_Cd_ molar ratios (0%: no addition; 4%: 400 µL; 8%: 800 µL; 12%: 1.2 mL). The reaction proceeded for an additional 12 min at 68.5°C before termination. The FTO/CdS samples were retrieved, ultrasonically cleaned in a 1:1 (v/v) ultrapure water/ethanol mixture for 2 min, rinsed with deionized water, and dried under N_2_ flow. Subsequently, 60 µL of 20 mg mL^−^^1^ CdCl_2_ in methanol was spin‐coated onto the films at 3000 rpm for 30 s, followed by atmospheric annealing at 400°C for 10 min. After natural cooling, samples were labeled as CdS:I‐0%, CdS:I‐4%, CdS:I‐8%, and CdS:I‐12% based on NaI added concentration.

### 4.2 Sb_2_Se_3_ Films Deposition by Chemical Bath Deposition

Sb_2_Se_3_ thin films were deposited on FTO/CdS substrates via chemical bath deposition (CBD). The reaction system comprised: 0.5 g antimony potassium tartrate (KSbC_4_H_4_O_7_·0.5H_2_O, Sb source), 2 mL of 0.1 mm sodium selenosulfate (Na_2_SeSO_3_, Se source), 20 mg selenourea (CH_4_N_2_Se, auxiliary additive), 10 mL ethanol (99.99%, cosolvent), and 28 mL ultrapure water (18.2 MΩ cm, solvent) in a 40 mL total volume. Pre‐treated FTO/CdS substrates were vertically immersed in the solution and maintained at 95°C for 150 min in a temperature‐controlled water bath. After deposition, samples underwent ultrasonic cleaning in deionized water (2 min) to remove residual complexes, followed by drying under high‐purity N_2_ flow (99.999%). All Sb_2_Se_3_ films were annealed at 375°C for 5 min under N_2_ atmosphere.

### 4.3 Sb_2_Se_3_ Based Solar Cell Device Fabrication

The hole‐transport layer (HTL) solution was formulated by mixing spiro‐OMeTAD (36.6 mg, Advanced Election Technology Co., Ltd.) as the host material in 1 mL of anhydrous chlorobenzene, with 4‐tert‐butylpyridine (tBP, 14.5 µL) and a Li‐TFSI solution (9.5 µL, 520 mg mL^−^^1^ in acetonitrile) as additives. This solution was spin‐coated onto the Sb_2_Se_3_ layer at 3000 rpm for 30 s using a volume of 50 µL. Subsequently, the coated substrates were stored in dry air for 24 h to allow for the oxidation of spiro‐OMeTAD. The devices were finalized by the thermal evaporation of an 80‐nm‐thick Au electrode through a shadow mask. The complete solar cells, with a structure of FTO/CdS/ Sb_2_Se_3_/spiro‐OMeTAD/Au, featured an active area of 0.04 cm^2^.

### 4.4 Characterization

Structural characterization of the Sb_2_Se_3_ films was performed by X‐ray diffraction (XRD, Empyrean) with a scanning range of 10.0° to 55.0°. The film morphologies were examined using a field‐emission scanning electron microscope (SEM, Hitachi S‐4800) at an acceleration voltage of 10.0 kV. Surface topography was obtained by atomic force microscopy (AFM, workshop TT‐AFM). The chemical composition and bonding states were analyzed by X‐ray photoelectron spectroscopy (XPS, VG Microtech ESCA2000) with a monochromatic Al‐Kα source operating at 250 W. Defect properties of the CdS films were studied by photoluminescence (PL) spectroscopy (Horiba Fluorescence FM). Optical properties were evaluated using a UV–vis‐near‐infrared spectrophotometer (UV/vis‐4802), with spectra collected from 300 to 1100 nm at a scan speed of 1200 nm min^−^^1^. The photovoltaic performance of the devices was evaluated by measuring current density–voltage (*J‐V*) curves under simulated AM 1.5 G solar irradiation (100 mW cm^−^^2^) using a Newport Oriel Sol3A solar simulator and a Keithley 2420 source meter. External quantum efficiency (EQE) was measured on a QTest Station 1000 ADI system in DC mode. For deep‐level transient spectroscopy (DLTS), devices were loaded into a Janis CCS‐150 cryostat, and measurements were conducted using a Zurich Instruments MFLI lock‐in amplifier in darkness, with the temperature stabilized by compressed helium gas and scanned from 480 K down to 100 K.

## Conflicts of Interest

The authors declare no conflicts of interest.

## Supporting information




**Supporting File**: advs74890‐sup‐0001‐SuppMat.docx.

## Data Availability

The data that support the findings of this study are available from the corresponding author upon reasonable request.
