# Supplementary material for: Diffusion‐Driven Targeted Passivation of Selenium Vacancies via an I‐Doped CdS Buffer Layer for Efficient Sb2Se3 Solar Cells
Source: Adv Sci (Weinh). 2026 Mar 20;13(31):e74890. doi: 10.1002/advs.74890 (PMC13588038; doi:10.1002/advs.74890)
Supplement: Supplementary file 1 [file ADVS-13-e74890-s001.docx]

**Diffusion-Driven Targeted Passivation of Selenium Vacancies via an I-Doped CdS Buffer Layer for Efficient Sb_2_Se_3_ Solar Cells**

Luyan Shen, ^1‡^ Deyang Qin,^2‡^ Er Nie,^1*^ Jiahua Tao^1^, Hidefumi Akiyama^3^, Junhao Chu^4^, and Shaoqiang Chen^1, 2*^

^1^Engineering Research Centre for Nanophotonics and Advanced Instrument, School of Physics and Electronic Science, East China Normal University, Shanghai, 200241, China;

^2^State Key Laboratory of Precision Spectroscopy, Department of Electronic Engineering, East China Normal University, Shanghai, 200241, China;

^3^Institute for Solid State Physics, The University of Tokyo, Kashiwanoha 5-1-5, Kashiwa, Chiba 2778581, Japan;

^4^Key Laboratory of Polar Materials and Devices, Ministry of Education, Department of Electronic Engineering, East China Normal University, 500 Dongchuan Road, Shanghai 200241, China.

**KEYWORDS**: Iodine doped CdS, Sb_2_Se_3_ thin films, Ion diffusion, defect passivation, solar cells.

‡ These authors contributed equally to this work.

**Email**: enie@phy.ecnu.edu.cn; [sqchen@ee.ecnu.edu.cn](mailto:sqchen@ee.ecnu.edu.cn).

**Note S1. Calculation details of texture coefficients.**

The Texture coefficients (TC) of the Sb_2_Se_3_ films are calculated by the following equation:

$TC\left( hkl \right)=\left( \frac{\frac{I_{\left( hkl \right)}}{I_{0\left( hkl \right)}}}{\frac{1}{n}\Sigma\frac{I_{\left( hkl \right)}}{I_{0\left( hkl \right)}}} \right)$	(S1)

where I_(hkl)_ and I_0(hkl)_ are the relative intensities of the (hkl) planes of the thin film and the standard powder sample (PDF:15-0861), respectively, and n is the total number of diffraction peaks used for evaluation. When TC(hkl)>1 signifies that the film grows along the given (hkl) crystal plane as the preferred orientation, while TC(hkl)<1 denotes that the orientation along the given (hkl) crystal plane is suppressed.

**Note S2.** Calculation method of conductivity ($\sigma_{0}$).

We can calculate the conductivity of CdS:I-0%, CdS:I-4%, CdS:I-8% and CdS:I-12% films according to Equation S2:

$I=\sigma_{0}Ad^{-1}V$	(S2)

where A and d represent the active area (0.04 cm^2^) of the sample and the film thickness, respectively. Form this, a large slope indicates a higher conductivity, and the $\sigma_{0}$ of CdS-0%, CdS-4%, CdS-8% and CdS-12% films are calculated to be 1.3×10^-3^, 1.6×10^-3^, 1.9×10^-3^ and 2.1×10^-3^ S m^-1^.

**Note S3. Calculation details of VBM, CBM, and E_F_.**

The conduction band minimum (CBM) and valence band maximum (VBM) of the Sb_2_Se_3_ films were calculated according to the following equations:

$E_{F}=21.22 eV-E_{cut-off}$	(S3)

$E_{VB}=-21.22-E_{cut-off}+E_{i}$	(S4)

$E_{CB}=E_{VB}+E_{g}$	(S5)

where E_F_, E_VB_, E_CB_, E_g_ are Fermi level, valence band maximum level, conduction band minimum level, and bandgap of film. *E_cutoff_* and *E_i_* represent the bandgap edge and tail obtain from UPS, respectively.

**Note S4. Calculation details of band gap of films.**

The band gap of Sb_2_Se_3_ films were calculated according to the Tauc equation:

$\alpha h\nu={A(h\nu-E_{g})}^{2}$	(S6)

where α and A are absorption coefficient and optical transition-dependent constant of the material under investigation, *hυ* is the energy of the photon, and *E_g_* is the band gap energy of the material.

**Note S5. Calculation details of DLTS measurement.**

The nature of the defects is characterized by deep-level transient spectroscopy (DLTS). The activation energy ($E_{a}$) and capture cross-section ($\sigma$) of a defect level can be determined from an Arrhenius plot, constructed using the relationship:

$\ln\left[ \frac{e\left( T \right)}{T^{2}} \right]=\ln\left( \gamma\sigma\right)-\frac{Ea}{kT}$	(S7)

where $e$ is the carrier emission rate, $T$ is the temperature, $k$ is the Boltzmann constant, and $\gamma$ is a constant. Fitting the data to this equation yields a straight line, whose slope is proportional to the activation energy $E_{a}$, and whose intercept is related to the capture cross-section $\sigma$. Furthermore, the trap density ($N_{t}$) can be calculated using the formula:

$N_{t}=2\frac{|\Delta c|}{c_{0}}N_{D}$	(S8)

where N_D_ is the net doping concentration in the space charge region, obtainable from C-V measurements. *c_0_* is the steady-state capacitance, and *Δc* is the trap-induced change in capacitance.

**Note S6. Density functional theory calculation details**

First-principles density functional theory (DFT) calculations were performed with the Perdew-Burke-Ernzerhof (PBE) exchange-correlation functional in a plane wave pseudopotential implementation using the Vienna ab initio simulation packages (VASP). A cutoff energy of 500 eV for the plane-wave basis set was employed. Energy and force convergence criteria were set as 1×10^-5^ eV and 0.02 eV/Å. The structural relaxation and density of states were calculated using 3×3×5 Gamma k-point meshes within the 40 atoms in one cell. The defect formation energy of defects (*E*_def_(*q*)) at different charge states *q* can be defined as Equation:

$E_{\mathrm{def}}(q)=E_{\mathrm{tot}}(\mathrm{defect})-E_{\mathrm{tot}}(\mathrm{pristine})-\mu_{I}+\mu_{X}+q(E_{F}-E_{\mathrm{VBM}})$	(S9)

Where $E_{\mathrm{tot}}(defect)$ is the total energy of the supercell containing the defect in charge state *q*, $E_{\mathrm{tot}}(pristine)$ is the total energy of the perfect Sb₂Se₃ supercell, $\mu_{I}$ denotes the chemical potential of the I atom (taken as half the total energy of solid-state I₂, $\mu_{I}=0.5E_{tot}\left( I_{2} \right)$), $\mu_{I}$ is the chemical potential of the substituted atom. The variable *q* indicates the charge state of the defect (covering possible ionization states with values of -1, 0, or +1), and $(E_{F}-E_{\mathrm{VBM}})$ represents the energy of the Fermi level relative to the valence band maximum (VBM), which ranges from 0 to 1.1 eV (corresponding to the bandgap of Sb₂Se₃). To ensure the stability of Sb₂Se₃, the chemical potentials must satisfy the constraint:

$2\mu_{Sb}+2\mu_{Se}=E_{tot}({Sb}_{2}{Se}_{3})/N$	(S10)where N is the number of Sb₂Se₃ formula units in the supercell.

**Note S7. Calculation details of internal defect density (N_trap_) based on SCLC.**

The defect density of Sb_2_Se_3_ can be calculation using the following Equation.

$N_{trap}=\frac{2\varepsilon\varepsilon_{0}V_{TEL}}{qL^{2}}$	(S11)

in which *ε* and $\varepsilon_{0}$ represent relative permittivity and vacuum permittivity (for Sb_2_Se_3_ film, $\varepsilon=13$ and $\varepsilon_{0}=8.854$×10^-12^ F m^-1^), respectively; $V_{TFL}$ is the threshold voltage for the trap-filled limit region and *L* is the film thickness; *q* is the elementary charge (1.602 × 10^-19^ C), L is the thickness of the Sb_2_Se_3_ film (279 nm).

**Note S8. Calculation details of carrier concentration (N_A_).**

The apparent carrier concentration (N_A_) was extracted from the slope of the 1/C^2^ vs V plot using the standard formula for an abrupt p-n junction:

$$\frac{1}{C^{2}}=\frac{2(V_{bi}-V)}{q\varepsilon_{0}\varepsilon_{r}N_{A}A^{2}}$$

Where *C* is the depletion-layer capacitance; $\varepsilon_{0}$ is the vacuum permittivity (8.854×10^-14^ F cm^-1^); $\varepsilon_{r}$ is the relative permittivity of the Sb_2_Se_3_ (19); *A* is the device’s cross-sectional area (0.04 cm^2^); *V* is the applied voltage; and q is the elementary charge (1.602×10^-19^ C).

**Figure S1.** The XRD pattern of FTO substrate.

**Figure S2.** The AFM images of CdS:I-0%, CdS:I-4%, CdS:I-8%, CdS:I-12%.

**Figure S3.** The grains distribution of CdS:I-0%, CdS:I-4%, CdS:I-8%, CdS:I-12%.

**Figure S4.** PL spectra of (a) CdS:I-4% and (b) CdS:I-12% films.

**Figure S5.** (a) The XPS of I 3d for the CdS:I-0% and CdS:F-8% films, respectively. (b) The XPS of Na 1s for the CdS:I-0% and CdS:I-8% films, respectively.

**Figure S6.** Thickness measurements of CdS:I-0%, CdS:I-4%, CdS:I-8%, CdS:I-12% films. All films with thickness about 70 nm are measured by Stylus Profiler.

Au/CdS:I-x%/Au devices were fabricated using approximately 70 nm thick CdS films for electrical characterization. The electrical conductivity gradually increased with higher iodine-doping concentration. The undoped CdS film exhibited a current of 0.068 A at 1 V, the corresponding conductivity is 1.3×10^-3^ S m^-1^. In contrast, the iodine-doped CdS:I film showed a significantly enhanced current output 0.093 A of CdS:I-4%, 0.108 A of CdS:I-8% and 0.1075A of CdS:I-12% at 1 V, respectively, the corresponding conductivity is 1.6×10^-3^ S m^-1^, 2.1×10^-3^ S m^-1^, 1.9×10^-3^ S m^-1^.This improvement in electrical conductivity helps reduce the series resistance in solar cells and enhances charge collection efficiency. The doping of I ions effectively optimize the window layer, leading to improved heterojunction quality and more efficient carrier extraction.

**Figure S7.** The HRTEM images of CdS:I/Sb_2_Se_3_ interface and corresponding cross-sectional EDS elemental mapping of CdS:I/Sb_2_Se_3_ device.

**Figure S8.** The texture coefficient of Sb_2_Se_3_ films based CdS:I-x% (x from 0% to 12%).

**Figure S9.** (a) UV-vis plots of CdS-Sb_2_Se_3_ and CdS:I-Sb_2_Se_3_. (b) Tacu plot of CdS-Sb_2_Se_3_ and CdS:I-Sb_2_Se_3_. (c) Valence band cutoffs and Fermi edges of UPS patterns of the CdS-Sb_2_Se_3_ film. (d) Valence band cutoffs and Fermi edges of UPS patterns of the CdS:I-Sb_2_Se_3_ film.

**Figure S10.** (a-b) Time-resolved absorption spectra obtained at various time delays after photoexcitation for the CdS/Sb_2_Se_3_ and CdS:I/Sb_2_Se_3_ thin films.

**Figure S11.** Space charge limited current (SCLC) measurements of CdS-Sb_2_Se_3_ film and CdS:I-Sb_2_Se_3_ film device.

**Figure S12.** The statistical boxplots of the photovoltaic parameters for the Sb_2_Se_3_ solar cells based on different I-doped CdS.

**Figure S13.** The stability test of CdS:I/Sb_2_Se_3_ devices in dry environment.

**Figure S14.** Nyquist plots of the CdS/Sb_2_Se_3_ and CdS:I/Sb_2_Se_3_ devices.

**Figure S15.** The band gap of CdS:I-0%, CdS:I-4%, CdS:I-8%, CdS:I-12%.

**Table S1.** Carrier lifetimes obtained from kinetic curves of CdS/Sb_2_Se_3_ and CdS:I/Sb_2_Se_3_ thin films.

|  | A_1_ | τ_1_ | A_2_ | τ_2_ | τ_ave_ |
| --- | --- | --- | --- | --- | --- |
| CdS/Sb_2_Se_3_ | 0.258 | 283 | 0.617 | 2393 | 2293 |
| CdS:I/Sb_2_Se_3_ | 0.580 | 362 | 0.446 | 4549 | 4156 |

**Table S2.** The band gap of CdS:I-0%, CdS:I-4%, CdS:I-8%, CdS:I-12%.

| Sample | CdS:I-0% | CdS:I-4% | CdS:I-8% | CdS:I-12% |
| --- | --- | --- | --- | --- |
| Band gap (eV) | 2.403 | 2.406 | 2.412 | 2.414 |
